# Optimizing droplet coalescence dynamics in microchannels: A comprehensive study using response surface methodology and machine learning algorithms

**DOI:** 10.1016/j.heliyon.2024.e41510

**Published:** 2025-01-03

**Authors:** Seyed Morteza Javadpour, Erfan Kadivar, Zienab Heidary Zarneh, Ebrahim Kadivar, Mohammad Gheibi

**Affiliations:** a Department of Mechanical Engineering, University of Gonabad, Gonabad, Iran; b Department of Physics, Shiraz University of Technology, Shiraz, 71555-313, Iran; c Institute of Ship Technology, Ocean Engineering and Transport Systems, University of Duisburg-Essen, 47057, Duisburg, Germany; d Institute for Nanomaterials, Advanced Technologies and Innovation, Technical University of Liberec, 46117, Liberec, Czech Republic; e Faculty of Mechatronics, Informatics, and Interdisciplinary Studies, Technical University of Liberec, Liberec, Czech Republic; f Faculty of Mechanical Engineering, Tarbiat Modares University, Tehran 141171-3116, Iran

**Keywords:** Coalescence, Droplet, Microchannel, Machine learning, Optimization

## Abstract

Droplet coalescence in microchannels is a complex phenomenon influenced by various parameters such as droplet size, velocity, liquid surface tension, and droplet-droplet spacing. In this study, we thoroughly investigate the impact of these control parameters on droplet coalescence dynamics within a sudden expansion microchannel using two distinct numerical methods. Initially, we employ the boundary element method to solve the Brinkman integral equation, providing detailed insights into the underlying physics of droplet coalescence. Furthermore, we integrate Response Surface Methodology (RSM) to effectively optimize droplet coalescence dynamics, harnessing the power of machine learning algorithms. Our results showcase the efficacy of these computational techniques in enhancing experimental efficiency. Through rigorous evaluation utilizing Regression Coefficient and Mean Absolute Error metrics, we ascertain the accuracy of our estimations. Our findings highlight the significant influence of key parameters, specifically the non-dimensional initial distance of the droplets (D), viscosity ratio (μ), Capillary number (Ca), and width (w), as identified by the non-dimensional final droplet-droplet spacing (DD), velocity of the first droplet (V_FD_), and velocity of the second droplet (V_BD_), respectively. This comprehensive approach provides valuable insights into droplet coalescence phenomena and offers a robust framework for optimizing microfluidic systems. The most influential parameters on DD are the values of A_d_ and D, while viscosity has the lowest influence on DD. The most influential parameters on droplet velocity are viscosity and channel width, whereas the initial distance and Ca have the least influence on droplet velocity. The comparison of different machine learning algorithms indicates that the best ones for predicting DD, V_FD_, and V_BD_ are function, SMOreg, Lazy-IBK, and Meta-Bagging, respectively.

**Table:** Nomenclature

		**Greek symbols**	
Aera	Area($m^{2}$)	μ	$\text{Vis}\cos\;i\text{ty}\;\text{ratio}\;\left(\mu_{c}/\mu_{d}\right)$
A	Non-dimensional Area (A = Area/w_1_^2^)	μ_c_	$\text{Vis}\cos\;i\text{ty}\;\text{of}\;\text{continous}\;\text{fluid}\left(N.s/m^{2}\right)$
Ca	Capillary Number	μ_d_	$\text{Vis}\cos\;i\text{ty}\;\text{of}\;\text{droplet}\left(N.s/m^{2}\right)$
d	The initial distance between two droplets(m)	ρ	$\text{Density}\left(\text{kg}/m^{3}\right)$
D	The non-dimensional initial distance between two droplets(d/w_1_)	**Subscript**	
dd	The final distance between two droplets(m)	1	Convergence part
DD	The non-dimensional final distance between two droplets(dd/w_1_)	2	Divergence part
l	Length (m)	FD	Front droplet
L	Non dimensional length (l/w_1_)	BD	Back droplet
v	Velocity (ms)		
V	Non-dimensional velocity $\left(\gamma /\mu_{c}\right)$		
w	Channel width(m)		
W	Non dimensional width (w/w_1_)		

## 1 Introduction

Microfluidics, an ever-evolving and dynamic field, is dedicated to comprehending, directing, and harnessing the behavior of fluids at the microscale. This interdisciplinary domain seamlessly blends principles from chemistry, physics, and engineering to scrutinize and manipulate fluid dynamics on a minute scale. At its core, microfluidics relies on ingenious devices designed to analyze, manipulate, and control fluid flow within tiny channels and chambers, enabling unparalleled precision and control over fluidic processes [1]. These microfluidic devices have found applications across various domains, including medical diagnostics, drug delivery, and chemical synthesis. Within the realm of microfluidics, captivating areas of research encompass droplet deformation [[2], [3], [4]], sorting [5,6], breakup [7,8], fusion [9,10], and the intriguing phenomenon of droplet coalescence [[11], [12], [13]].

Droplet coalescence, a pivotal phenomenon in microfluidics, involves the fusion of two or more small droplets into a larger, unified droplet [14,15]. This process holds immense significance in both natural phenomena and industrial applications, where tiny droplets converge, such as in raindrop formation, emulsion stability, chemical reactions, and the functioning of microfluidic devices [16,16,17,17,18,18]. The coalescence process is governed by the interplay of attractive forces among the droplets, which ultimately triumph over the surface tension forces that endeavor to keep them apart. When two droplets approach one another, their surfaces undergo deformation and gradually meld, giving rise to a thinning connection or 'neck' between them [19,20]. With time, this neck widens, allowing the liquid from one droplet to seamlessly flow into the other, culminating in the amalgamation of the two into a single, larger droplet [21]. Understanding droplet coalescence is not merely about elucidating a physical process; it unveils a captivating microcosm where the delicate dance of forces and fluids at the microscale yields invaluable insights and applications across a diverse spectrum of fields. In essence, the study of microfluidics, and more specifically, droplet coalescence, serves as a window into the intricate interplay between forces and fluids, offering opportunities for advancements in various scientific and technological endeavors.

A multitude of factors intricately influences the rate and extent of droplet coalescence, each contributing to the intricate dance of tiny liquid entities. These factors include the surface tension properties of the involved liquids, the size and shape of the droplets, the ratio of viscosities, temperature, the presence of surfactants, and the geometry of the microfluidic channels [22]. Understanding how these factors interact and influence droplet coalescence dynamics is essential for optimizing processes in microfluidic devices and harnessing their full potential in various applications.

In specific scenarios, droplet coalescence is a sought-after phenomenon, such as in the creation of stable emulsions. In this context, it is essential to merge immiscible liquids into a harmonious mixture [22]. However, there are instances, such as in the case of oil spills or inkjet printing, where droplet coalescence can be highly undesirable. In such situations, the merging of distinct droplets diminishes the efficiency and effectiveness of the process [23,24]. The geometry of the microfluidic channel itself plays a pivotal role in influencing droplet coalescence dynamics. For instance, Schirrmann et al. introduced the concept of a sudden expansion channel to explore the intricacies of droplet coalescence within such microfluidic configurations. Their experimental work delved into the effects of varying width ratios (width of the sudden expansion channel to the width of the narrow channel) on droplet coalescence [25]. Drawing inspiration from their research, Kadivar et al. undertook a numerical investigation into the dynamics of droplet coalescence within the sudden expansion channel [25,26]. Ferrás et al. delved into the effects of slip and no-slip boundary conditions on droplet coalescence within this microchannel context [27]. Expanding the horizons of investigation, Christopher et al. conducted experimental studies to explore the influence of carrier flux and droplet size on the dynamics of droplet coalescence in T-junction microfluidic channels [28]. Deng et al. [29] proposed a T-junction microfluidic channel with a lantern-shaped expansion to further probe the dynamics of droplet coalescence, with their experimental results highlighting how the film drainage time of the carrier liquid decreases as flow velocity and droplet size increase. In a different avenue of exploration, Yang et al. scrutinized the dynamics of droplet coalescence within T-shaped junctions, uncovering three distinct coalescence regimes that hinged on the viscosity ratio and flow rate ratio [30]. Meanwhile, researchers have also explored the effects of parameters such as the capillary number and channel intersection angle on droplet coalescence in Y-junction microfluidic channels. Their experimental findings indicate that the film drainage time decreases with increasing flow rate and Y-junction angle, shedding light on yet another facet of this intricate phenomenon [31].

Williams et al. [32] delved into the intricacies of oil-in-water coalescence kinetics using a droplet number density approach. Their investigation centered on scrutinizing the impact of various factors, such as the ratio of droplet sizes, phase volume fraction, and viscosity ratio, on the coalescence kinetics of oil-in-water systems. Notably, Blanchete et al. [33] conducted a comprehensive exploration into the role of surface tension in droplet coalescence within a reservoir, employing both experimental and numerical methodologies. Their research uncovered insights into how droplet size and reservoir composition influence the type of coalescence observed. Meanwhile, Dudek et al. [34] dedicated their efforts to understanding the behavior of crude oil droplet coalescence in the water phase. Their findings revealed that larger droplets exhibited slower coalescence rates, contributing to a reduction in coalescence frequency.

An alternative approach to droplet coalescence involves the application of a thermal field, where integrated microheaters situated outside of microchannels generate this thermal environment. Luong et al. [35] carried out experimental investigations into the coalescence of two droplets induced by temperature changes within a microfluidic channel. Temperature-induced droplet coalescence in microchannels also drew the attention of Xu et al. [36], who employed numerical methods, including finite volume and level set techniques, to dissect the dynamics of droplet coalescence. These studies illuminated the dependence of droplet coalescence on the temperature of the microfluidic chip. Intriguingly, experiments conducted across a temperature range of 20–70 °C yielded results that showcased an increase in droplet coalescence frequency with rising temperature [37].

Dudek et al. [34] introduced a novel microfluidic methodology capable of analyzing over a hundred droplets traversing a microfluidic channel. As they increased the velocity of the continuous phase, they observed a reduction in both contact time and coalescence time, ultimately reaching critical values. Wang et al. [38] ventured into examining the effect of interfacial gas enrichment on droplet coalescence, and their experiments unveiled that the attraction between droplets and the continuous phase interface of foam films could be finely controlled through the injection of oxygen gas. Politova et al. [24] investigated the dependency of droplet coalescence stability on variables like droplet size, surfactant concentration, and fluid viscosity. Their research unearthed that higher surfactant concentrations significantly bolstered the stability of larger water droplets. Furthermore, Wang et al. [39] harnessed the method of volume of the fluid to probe the stability of two droplets during coalescence within microfluidic channels, exploring the influence of the initial droplet size on deformation and coalescence time.

In a cross-sectional microfluidic channel context, Yi et al. [40] made noteworthy observations, identifying four distinct coalescence regimes. Their research also pointed to a reduction in liquid film drainage time as the velocity of the carrier liquid increased. Furthermore, they conducted experiments to study the interfacial evolution of droplet coalescence in straight channels with varying height-to-width ratios, finding that the width of the liquid bridge expanded in response to higher flow rates of the droplet phase relative to the carrier phase [41].

Combining the high-throughput capabilities of droplet microfluidics (DM) with the analytical power of machine learning (ML) significantly enhances droplet-based microfluidic technology. This synergy enables the development of optimized and automated microfluidic systems that offer greater accuracy, reduced processing times, and minimal need for human intervention.

Some researchers focused on the prediction of droplet size and shape. Mahdi and Daoud [42] employ artificial neural networks to forecast the sizes of microdroplets in water-in-oil emulsions within microfluidic systems. They presented a best-performing neural network model that demonstrates both low mean square error and high accuracy, with the significance of its inputs confirmed using the Garson algorithm. The ANFIS model is used to predict the droplet size with an accuracy of 96 % and 92 %, respectively [43,44]. Also, Srikanth et al. [45] applied the ANN algorithm to predict the diameter of the droplet in a microfluidic device and extracted a model with an R_2_ value of 0.97.

Wikramanayake and Bahadur [46] utilized statistical modeling to forecast the coalescence behavior of water droplets in an electrowetting (EW) field. They found that applied voltage and electrode geometry play a crucial role in droplet coalescence, whereas AC frequency has no significant effect. Angardi et al. [47] examined the coalescence of single droplets at the oil-water emulsion interface through statistical analysis, focusing on droplet size, viscosity, and aqueous phase composition. They identified relationships between coalescence time and controllable variables such as continuous phase viscosity, droplet size, interfacial tension, and densities of both phases. Their findings showed that coalescence time increases with higher surfactant concentration and continuous phase viscosity. Hu et al. [48] employed machine learning models (Random Forest, XGBoost, and Multilayer Perceptrons) to predict droplet coalescence in microfluidic devices. They demonstrated that Random Forest's ensemble approach provides resilience against missing key features, whereas XGBoost's boosting method increases its sensitivity to such gaps.

Recently, droplet coalescence within a sudden expansion channel has been investigated [[25], [26], [27]]. Additionally, some researchers have studied droplet coalescence within different geometries, such as T-junctions [[28], [29], [30], [31],^49^]. Furthermore, a few studies have been carried out to predict droplet size [43,44,50] or behavior droplet coalescence [48] in microfluidic systems using machine learning or statistical analysis [46,47]. However, this study investigated the condition of flow and droplets in the sudden expansion channel to predict the phenomena of coalescence. Additionally, using the response surface method and machine learning algorithms, the effects of different parameters on droplet velocity and droplet coalescence were extracted and optimized.

In our current research, we address the dynamic intricacies of droplet coalescence within a sudden expansion channel by combining the boundary integral equation of the Brinkman differential equation with cutting-edge machine-learning techniques. Our study fills crucial research gaps by offering a multifaceted approach. Initially, we apply the boundary integral method to transform the Brinkman differential equation into a self-consistent integral equation, enabling a comprehensive understanding of droplet behavior. Subsequently, utilizing the boundary element method, we effectively calculate the velocities of both droplets and the carrier liquid, providing detailed insights into coalescence dynamics.

Moreover, our research extends into the domain of smart systems by employing machine learning algorithms, i.e., Lazy IBK, SMOreg, and Meta Bagging, as soft sensors for experimental outputs. These algorithms predict essential outputs derived from practical experiments, significantly enhancing the predictive capabilities of our research and addressing the need for robust modeling techniques in microfluidics.

In our ongoing research endeavors, we emphasize optimizing the effective features utilized in experimental practices. Leveraging the Response Surface Methodology (RSM) technique, we engage in experimental design and optimization, ensuring the efficiency and reliability of our procedures. Through sensitivity analyses, mathematical modeling, and regression-based optimization techniques, we extract valuable insights, bridging the gap between theoretical understanding and practical implementation in microfluidic systems.

In conclusion, our research offers a comprehensive approach to understanding and optimizing droplet coalescence dynamics in microchannels. By integrating numerical methods, machine learning algorithms, and experimental optimization techniques, we contribute to advancing the field of microfluidics, paving the way for enhanced control and efficiency in various applications.

## 2 Physical model and numerical method

**Fig. 1:**
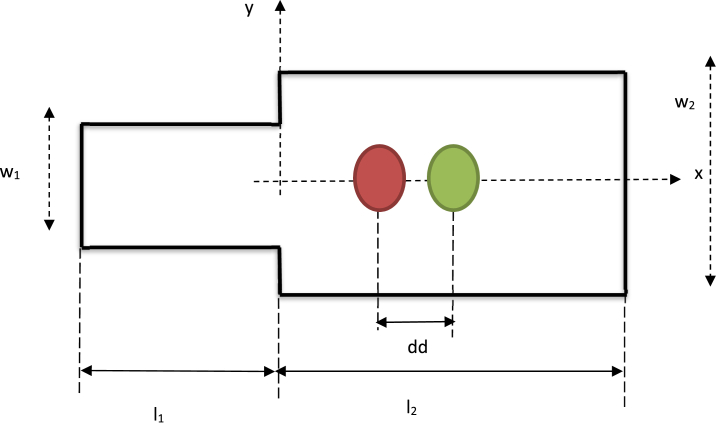
Sketch of studied microchannel.

We conducted a numerical investigation into the coalescence dynamics of flattened, disk-like deformable droplets within a microfluidic channel. This channel comprises two straight sections with differing widths, denoted as W_1_ and W_2_, where W_2_ is greater than W_1_. The narrower straight channel connects to the wider channel through two right-angle turns, as depicted in Fig. 1. To provide a consistent scale, all lengths are normalized with respect to the width of the upstream channel, W_1_. Our analysis is carried out in a two-dimensional Cartesian coordinate system, where the microchannel's centerline is represented by y = 0, and the inlet of the sudden expansion section is designated as x = 0. The inlet and outlet of the microchannel are positioned at X = −20 and X = 20, respectively. Along the narrow straight channel (x < 0), the lower and upper walls correspond to Y = −1/2 and Y = 1/2, respectively. In the downstream segment of the channel (x > 0), the lower and upper walls are situated at Y = − w_2_/w_1_ and Y = w_2_/w_1_, respectively. For consistency, we scale all time, distance, and velocity parameters according to:

(1) $$T=\frac{\mu_{c\;}w_{1}}{\gamma },W=\frac{w}{w_{1}},L=\frac{l}{w_{1}},V=\frac{\gamma }{\mu_{c}},A=\frac{\text{Area}}{w_{1}^{2}},\mu =\frac{\mu_{c}}{\mu_{d}},\text{DD}=\frac{\text{dd}}{w_{1}},D=\frac{d}{w_{1}}$$

Where γ represents the interfacial tension, additionally, the capillary number is employed to describe the ratio of viscous forces to surface tension forces within the system(equation (2)).

(2) $$\text{Ca}=\frac{\mu_{c}v}{\gamma }$$

The non-dimensionless Brinkman and mass conversion equations are given by:

(3) $$\nabla P^{\left(i\right)}=\alpha_{i}\left(\nabla^{2}V^{\left(i\right)}-k^{2}V^{\left(i\right)}\right)\;on\;\Omega_{i}\;with\;i\in \left\{c,d\right\}\text{,}$$

(4) $$\nabla ∙V^{\left(i\right)}=0\;on\;\Omega_{i}\;with\;i\in \left\{c,d\right\}\text{.}$$

In the provided equations, the symbol P(x,y) represents the dimensionless pressure, while V represents the depth-averaged dimensionless velocity. Here, the parameter k is defined as the square root of 12 divided by *H* (channel height) and $\alpha_{i}$ denotes the ratio of the dynamic viscosity of phase i (*μ*_*i*_) to the dynamic viscosity of the continuous phase (*μ*_*c*_). The pressure boundary condition at the interface between the two phases dictates that the normal component of the stress tensor is discontinuous across this interface. This discontinuity in the normal stress at the droplet interface has been documented in prior research [51].

(5) $$\sigma^{\left(d\right)}\cdot n-\sigma^{\left(c\right)}\cdot n=\gamma \left(\frac{\pi }{4}\;\kappa_{∥}+\kappa_{⊥}\right)n$$

In the provided context, the term σ⋅n signifies the normal component of the stress tensor, where the unit normal director, n, is oriented from the droplet interface into the carrier liquid. Additionally, *κ*_*∥*_ and *κ*_*⊥*_ represent the curvatures of the droplet interface parallel and perpendicular to the flow direction. It's important to note that, based on the mass conservation equation, the velocity of the droplet at the droplet interface is set equal to the velocity of the carrier fluid. This condition is also imposed on all solid walls within the system. Furthermore, at the inlet of the narrow straight microchannel, we consider the Darcy-Brinkman velocity as described in Ref. [52].

(6) $$V_{in}^{\left(1\right)}\left(y\right)=Ca\frac{\cosh\left(kW_{1}/2\right)-\cosh\left(ky\right)}{\cosh\left(kW_{1}/2\right)-1}$$

Where Ca denotes the capillary number.

In our current investigation, we compute the x and y components of velocity on the droplet interface using the boundary element method. This method involves the transformation of the Brinkman partial differential equation into an integral equation. In accordance with the principles outlined in the boundary element method [49,53], the j-component of the local velocity of the carrier liquid is determined as follows:

(7) $$V_{j}\left(r_{0}\right)=\frac{1}{C_{l}}\oint_{\Gamma_{w},\Gamma_{O}}\left[V_{i}\left(r\right)T_{ijk}^{B}\left(r,r_{0}\right)n_{k}-\sigma^{\left(c\right)}n_{i}G_{ij}^{B}\left(r,r_{0}\right)\right]dl+\frac{1}{C_{l}}\oint_{{\Gamma_{c}}_{d}}\left[\left(\alpha_{d}-1\right)V_{i}\left(r\right)T_{ijk}^{B}\left(r,r_{0}\right)n_{k}-\gamma \left(\frac{\pi }{4}\kappa_{∥}+\kappa_{⊥}\right)n_{i}G_{ij}^{B}\left(r,r_{0}\right)\right]dl\text{,}$$

r=(x.y) denotes the field point and $r_{0}=\left(x_{0}.y_{0}\right)$ calls the singular point. If the singular point is located on the solid walls or droplet interface, then $C_{l}=\frac{1}{2}$ or $C_{l}=\frac{1+\alpha_{d}}{2}$ , respectively. $\Gamma_{cd}$ and $\Gamma_{w}$ are the droplet and wall contours, respectively. $\Gamma_{o}$ is integration path of the inlet and outlet of the microfluidics. $G_{ij}^{B}$ and $T_{ijk}^{B}$ are velocity and stress tensor Green's functions of Brinkman's equation, respectively. Velocity and stress tensor Green's functions read [44,45]

(8) $$G_{ij}^{B}\left(r.r_{0}\right)=-\delta_{ij}A\left(k\rho \right)+\frac{i_{j}}{\rho^{2}}B\left(k\rho \right)\text{,}$$

(9) $$\begin{array}{rrr} T_{ijk}^{B} & = & \delta_{ij}\frac{\rho_{j}}{\rho^{2}}2\left[B\left(k\rho \right)-1\right]+\frac{\delta_{ijk}+\delta_{kji}}{\rho^{2}}C\left(k\rho \right)\\ & & -4\frac{i_{j}j_{k}}{\rho^{4}}D\left(k\rho \right)\text{,} \end{array}$$

Where we have:

(10) $$\begin{array}{r} A\left(\rho \right)=2\left[\frac{1}{k^{2}\rho^{2}}-\frac{K_{1}\left(k\rho \right)}{k\rho }-K_{0}\left(k\rho \right)\right]\text{,}\\ B\left(\rho \right)=2\left[\frac{2}{k^{2}\rho^{2}}-2\frac{K_{1}\left(k\rho \right)}{k\rho }-K_{0}\left(k\rho \right)\right]\text{,}\\ C\left(\rho \right)=\frac{8}{k^{2}\rho^{2}}-4K_{0}\left(k\rho \right)-2\left(k\rho +\frac{4}{k\rho }\right)K_{1}\left(k\rho \right)\\ D\left(\rho \right)=C\left(k\rho \right)+k\rho K_{1}\left(k\rho \right) \end{array}$$

The symbols used in our equations are as follows: represents the vector difference between the field point ***r*** and the collocation point ***r***_*0*_, *ρ* is the magnitude of , and $K_{0}\left(k\rho \right)$, and $K_{1}\left(k\rho \right)$ are modified Bessel functions. It's worth noting that the integrands exhibit singularity treatment as the field point r approaches the collocation point r_0_.

In line with the boundary element method, we discretize all boundaries into finite boundary elements. Specifically, the solid channel walls, outlet, and inlet domains are divided into straight elements. For the deformable droplets, we employ cubic-spline elements for discretization. We also enforce periodicity conditions for the first and second derivatives at both the first and last nodes. The coordinates of cubic spline elements are represented in terms of cubic polynomials. To ensure grid independence, we assess the dimensionless droplet area as a function of time. The droplet area needs to remain constant as it flows through the microchannel. Our numerical results indicate that employing 120 cubic-spline elements for the droplet interface and 388 straight elements for solid walls, inlet, and outlet is sufficient. Increasing the number of elements beyond these values does not yield significant changes in the numerical results.

To discretize the self-consistent integral equation (7), we utilize the boundary element collocation method. The integrals are evaluated using Gauss-Legendre quadrature with 12 nodes over each element, resulting in a dense N × N linear system of algebraic equations. This linear system is subsequently solved using the Gaussian elimination method.

The advancement of the droplet through time is accomplished using the explicit Euler scheme, where discrete time steps are applied.

(11) $$x^{\left(n+1\right)}=\int_{t=n}^{t=n+1}V_{x}dt+x^{\left(n\right)},y^{\left(n+1\right)}=\int_{t=n}^{t=n+1}V_{y}dt+y^{\left(n\right)}\text{,}$$

The local velocity, denoted as V, is computed based on the boundary element problem equation (7) using the nodes located at r_0_. It's important to note that as time progresses, the distance between two neighboring points on the droplet contour changes. Consequently, it becomes imperative to mesh the droplet interface after each time step. This meshing of the droplet contour is achieved through the utilization of a cubic interpolation method.

## 3 Modelling processes

### 3.1 Response surface method(RSM)

A well-considered experimental design is implemented to explore the interplay of factors systematically. This includes a central composite design (CCD) matrix encompassing different levels of the factors in question. These levels span low, intermediate, and high settings, as well as center points designed to assess measurement error and variability. In the data collection and analysis phase, experiments are meticulously executed according to the predetermined CCD matrix. Measurements of DD, V_FD_, and V_BD_ are meticulously recorded for each unique combination of factor settings. This empirical data subsequently forms the basis for constructing response surface models that establish the intricate relationships between the factors and the responses. These models encompass not only linear and quadratic terms but also interaction terms, thereby capturing the diverse influences of the factors on the responses.

The optimization procedure leverages the desirability function approach to optimize multiple responses concurrently. This approach facilitates the identification of factor levels that lead to the maximization or minimization of the responses, accounting for their relative importance. Employing numerical optimization techniques, such as gradient-based methods, the optimal factor settings are determined, reflecting the ideal conditions for attaining the desired DD, V_FD_, and V_BD_ values.

In this part, we identify the key factors using analysis of variance (ANOVA) and determine the optimal levels for adjusting each output by applying the signal-to-noise ratio (SNR) [54]. Model validation constitutes a pivotal step in ensuring the robustness of the methodology. A subset of experiments is performed using the optimized factor levels to validate the accuracy of the response surface models and the efficacy of the optimization process. Statistical analyses, encompassing methods like analysis of variance (ANOVA) and residual analysis, serve as essential tools to assess the validity of the models and the reliability of the optimization outcomes [[55], [56]]. Moreover, a sensitivity analysis is conducted to evaluate the varying impact of effective features (W, μ, A_d_, Ca, and D) on the responses of interest. This analysis elucidates the relative importance of each factor in influencing DD, V_FD_, and V_BD_. In this research, Design Expert 7.0.0 software is applied due to the implementation of optimization practices. Likewise, all the applied data and their limitations are mentioned in Table 1.

**Table 1:** The available data of RSM computations in this study.

**No**	**W**	μ	**A**_**d**_	**Ca**	**D**	**DD**	**V**_**FD**_	**V**_**BD**_
**1**	5.269	3	1.936	0.0996	0.395	0.408	0.354	2.624
**2**	5.695	1.823	0.849	0.0767	0.425	0.516	0.86	1.142
**3**	5.333	2.733	1.982	0.098	0.327	0.336	0.450	2.072
**4**	5.997	1.252	0.841	0.071	0.373	0.471	1.075	0.908
**5**	5.295	2.781	1.919	0.092	0.379	0.395	0.436	2.149
**6**	5.609	1.448	1.922	0.067	0.341	0.370	0.933	1.019
**7**	5.269	2.873	1.936	0.099	0.395	0.408	0.400	2.333
**8**	5.963	1.483	0.937	0.066	0.409	0.504	0.985	0.992
**9**	5.323	2.953	1.681	0.081	0.367	0.397	0.389	2.445
**10**	5.522	2.416	1.851	0.082	0.385	0.412	0.58	1.633
**11**	5.852	2.042	1.69	0.073	0.404	0.452	0.733	1.302
**12**	5.981	1.313	0.795	0.063	0.439	0.544	1.056	0.927
**13**	5.28	2.95	1.946	0.099	0.349	0.359	0.372	2.5
**14**	5.865	1.847	1.508	0.086	0.399	0.455	0.814	1.179
**15**	5.814	2.583	1.312	0.073	0.368	0.431	0.556	1.741
**16**	5.34	2.710	1.858	0.081	0.329	0.349	0.468	2.01
**17**	5.612	1.362	1.038	0.075	0.435	0.517	1.016	0.96
**18**	5.234	2.831	1.96	0.088	0.380	0.393	0.415	2.255
**19**	5.68	2.275	0.816	0.069	0.429	0.522	0.696	1.419
**20**	5.924	1.903	1.678	0.070	0.405	0.456	0.786	1.215
**Max**	**5.997**	**2.998**	**1.982**	**0.099**	**0.434**			
**Min**	**5.234**	**1.253**	**0.795**	**0.063**	**0.327**			
**Average**	**5.593**	**2.229**	**1.523**	**0.081**	**0.387**			

### 3.2 Machine learning computations

In this section, we describe the methodology employed for implementing a smart system using machine learning techniques. Specifically, we apply the Lazy-IBK (Instance-Based Learning with k Nearest Neighbors), SMOreg (Sequential Minimal Optimization for Regression), and Meta Bagging algorithms in the WEKA version 3.9 software environment. The objective is to tune these models and assess their statistical performance using k-fold cross-validation, ranging from 2 to 10 folds with 2-fold intervals. The evaluation of model efficiency is based on two key indicators: Regression Coefficient (RC) and the Mean Absolute Error (MAE).

The Lazy algorithm is an instance-based learning approach that makes predictions based on the similarity of instances in the training dataset. For regression tasks, the prediction for a new instance is computed as the weighted average of the target values of its k-nearest neighbors (Equation (12)).

(12) $$\hat{y}=\frac{1}{k}\sum_{i=1}^{k}y_{i}$$

$\hat{y}$ is the predicted target value.
y_i_ is the target value of the i-th nearest neighbor.
IBK is a variation of the Lazy algorithm that uses the k-nearest neighbors to make predictions. It follows the same mathematical formulation as the Lazy algorithm.

SMOreg is a support vector regression algorithm that aims to find a hyperplane that best fits the training data. The prediction for a new instance is calculated as the dot product between the input features and a weight vector plus a bias term (Equation (13)).

(13) $$\hat{y}=\sum_{i=1}^{n}\alpha_{i}\;y_{i}\;\left(x,x_{i}\right)+b$$

where.

y^^^ is the predicted target value.
y_i_ is the target value of the i-th support vector.
n is the number of support vectors.
b is the bias term.
x is the input feature of the new instance.
x_i_ is the input feature of the i-th support vector.
α_i_ are the Lagrange multipliers.

Meta-bagging is an ensemble learning technique that combines multiple base models to improve prediction accuracy. It employs bootstrapped subsets of the training data to train individual models and then aggregates their predictions.

To evaluate the performance of the machine learning models, we employ k-fold cross-validation with varying values of k, ranging from 2 to 10 with a 2-fold interval. For each fold, the following evaluation metrics are calculated:

The RC measures the strength and direction of the linear relationship between the predicted and actual target values. It is calculated using the following formula:

(14) $$RC=\frac{COV\left(y,\hat{y}\right)}{\sqrt{Var\left(\hat{y}\right)-Var\left(y\right)}}$$

The Mean Absolute Error (MAE) measures the average absolute difference between the predicted and actual target values. The following formula calculates the MAE:

(15) $$MAE=\frac{1}{N}\sum_{i=1}^{N}\left(y_{i}-\hat{y}\right)$$

Where N is the number of instances.

## 4 Results and discussions

**Fig. 2:**
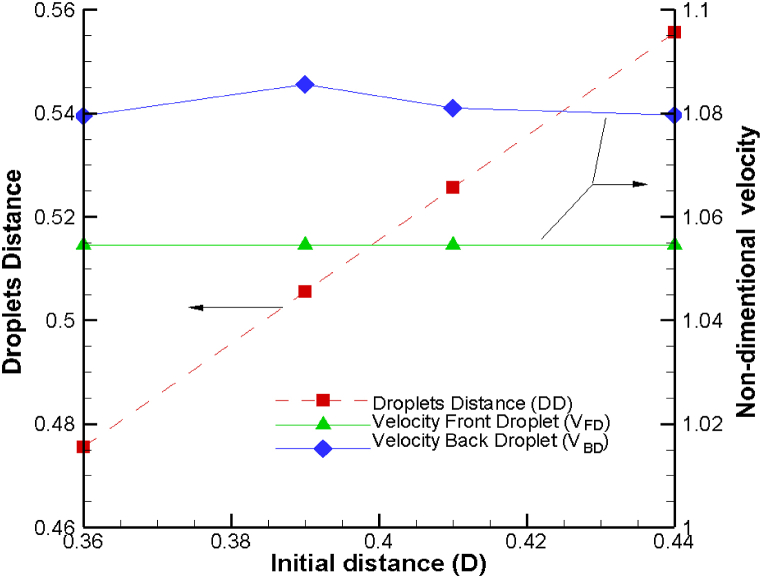
Variation of droplet velocity at the section of the sudden expansion of channel and droplet-droplet distance as a function of initial distance on droplet distance.

In Fig. 2, we present a comprehensive view of the dynamics involving dimensionless droplet-droplet distances and the dimensionless droplet velocities of both front and back droplets. These dynamics are examined concerning the initial droplet-droplet distance, specifically when the back droplet is positioned at the entrance of the sudden expansion region. A noteworthy observation is that the dimensionless droplet velocity at the entrance point of the sudden expansion channel remains consistent regardless of the initial droplet-droplet distance. However, it's important to emphasize that several key factors, including the capillary number, viscosity ratio, droplet area, and channel geometry, influence the overall droplet velocity. These parameters collectively determine the droplet's behavior within the channel.

Additionally, as the initial droplet-droplet distance in the narrow channel increases, there is a noticeable trend of the droplet-droplet distance also increasing. Interestingly, in response to increasing the initial droplet distance, the droplets' velocities exhibit a relative constancy, while the distance between them exhibits a linear change. This behavior provides valuable insights into the interplay between initial conditions and the evolving dynamics of droplet interactions within the microchannel.

To investigate the impact of droplet area on our objective function, we maintain the initial droplet distance, capillary number, and viscosity ratio as constants while varying the droplet area. Notably, within the sudden expansion region, we observe a significant phenomenon: the carrier flow streamlines diverge as they approach the opening. As a result, larger droplets encounter higher shear forces along the y-axis, causing them to elongate in the y-direction. Consequently, an increase in droplet area leads to a decrease in droplet velocity.

**Fig. 3:**
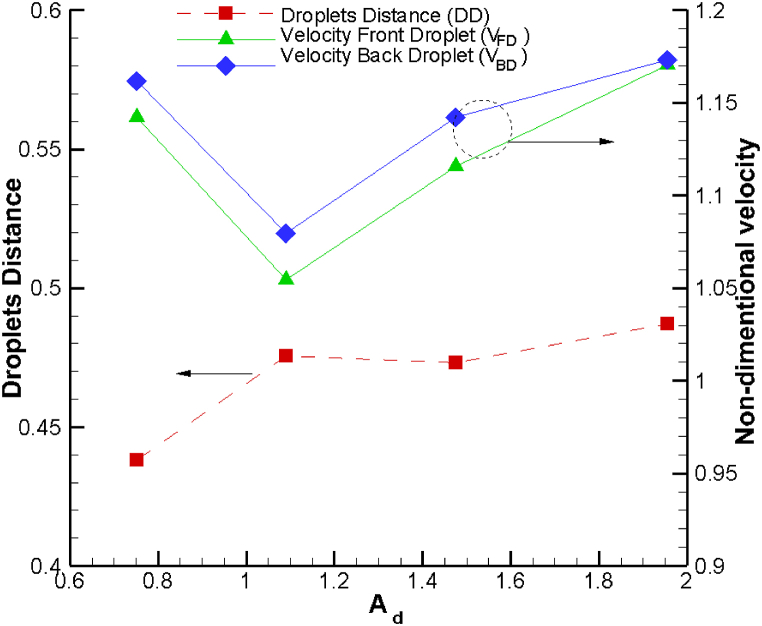
Droplet velocity at the section of the sudden expansion of channel and droplet-droplet distance as a function of droplet area.

Fig. 3 provides a visual representation of the droplet-droplet distance within the sudden expansion channel as a function of droplet area, with the back droplet positioned at the channel's opening. Interestingly, at A_d_>1, we observe that the droplet-droplet distance remains nearly constant with increasing droplet area. Furthermore, we identify two distinct behaviors for droplet velocity at the opening of the sudden expansion channel concerning droplet size.

When the droplet diameter is smaller than the upstream channel width, W_1_, an increase in droplet size results in a decrease in droplet velocity at the opening point. However, in cases where the droplet diameter surpasses the upstream channel width, the droplet velocity increases as the droplet size grows. This dual behavior underscores the intricate relationship between droplet size and the dynamics within the microchannel, shedding light on how different size configurations influence the velocity of the droplets at the opening point of the sudden expansion region. As the droplet area increases up to (A_d_ < 1.1), the distance between droplets increases by up to 12 %. Beyond this point, the changes become insignificant.

**Fig. 4:**
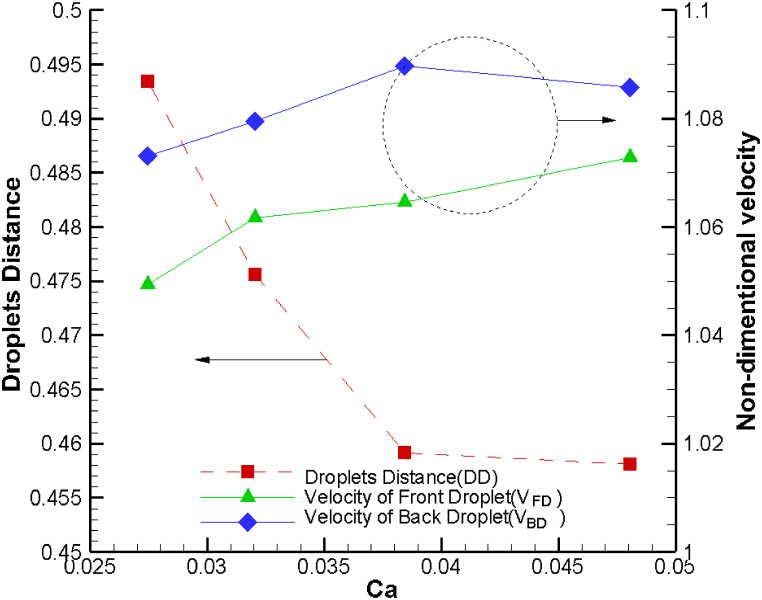
Variation of droplet velocity at the section of the sudden expansion of channel and droplet-droplet distance as a function of capillary number.

Another crucial parameter influencing both droplet-droplet distance and droplet velocity is the capillary number. To explore the impact of the capillary number on these dimensionless variables, we maintained a constant inlet flow rate while varying the surface tension. Consequently, as we modified the surface tension, we observed that the velocities of both droplets remained relatively constant, regardless of changes in the capillary number. Fig. 4 provides a visual representation of the variations in droplet-droplet distance and droplet velocity with respect to the capillary number. It's essential to note that as surface tension decreases, the capillary number increases. This decrease in surface tension leads to enhanced droplet deformation at the opening of the sudden expansion channel. Essentially, the front droplet elongates along the perpendicular axis to the flow direction and spends more time within the diverging streamline region.

Consequently, this extended deformation allows the back droplet additional time to approach the front droplet. Consequently, the droplet-droplet distance at the opening region decreases, highlighting the intricate interplay between surface tension, capillary number, and droplet dynamics within the microchannel. However, with an increasing capillary number, droplet-droplet distance is decreased by 8 %.

The dynamic viscosity ratio is defined as the viscosity ratio of the droplet phase to the carrier phase. In this study, our focus is on understanding how variations in the dynamic viscosity ratio impact our objective functions. To isolate the effects of the dynamic viscosity ratio, we maintain all other input parameters, such as droplet size, capillary number, and initial droplet distance, at constant values and solely modify the viscosity ratio. It's important to note that this variation in the viscosity ratio should be achieved without affecting the capillary number. We keep the viscosity of the carrier phase fixed while altering only the droplet viscosity.

**Fig. 5:**
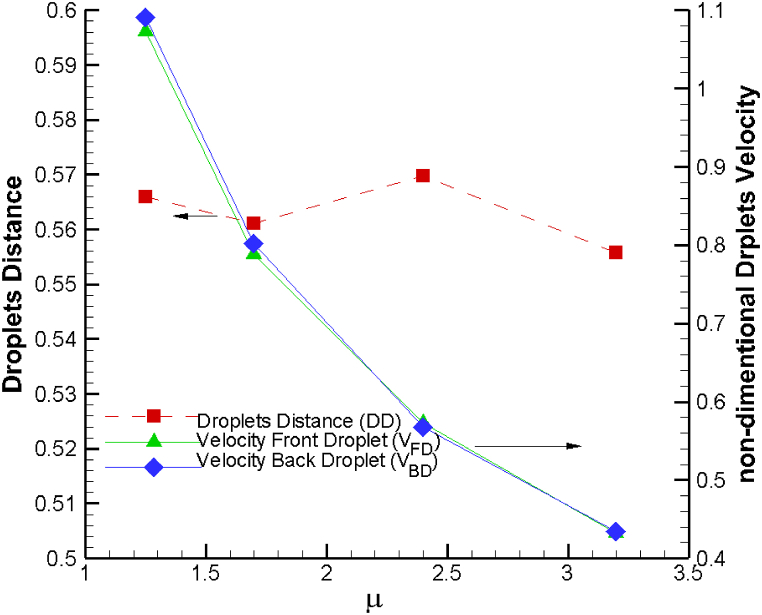
Droplet velocity at the section of the sudden expansion of channel and droplet-droplet distance as a function of viscosity ratio of droplet phase to continuous phase.

Fig. 5 provides a visual representation of how changes in the viscosity ratio affect droplet velocity and droplet-droplet distance. As is widely understood, the drag force exerted on the droplet surface is highly dependent on the viscosity ratio. When the dynamic viscosity ratio is increased, the drag force experienced by the droplet likewise increases. Consequently, this heightened drag force leads to a reduction in droplet velocity as the viscosity ratio of the droplet phase to the carrier phase increases. These findings shed light on the pivotal role of viscosity ratios in influencing the velocity and interactions of droplets within the microchannel. The viscosity ratio does not significantly affect the droplet distance. However, an increase in viscosity ratio reduces the velocity of droplets by 16 %.

### 4.1 RSM model execution

In this study, different mathematical models are applied due to curve fitting of the 3-D models based on three functions, including DD, V_FD_, and V_BD_. The results demonstrated that in all the mentioned functions, the Second-Order Factorial Interactions (2FI) model is selected as the best model. The RC/predicted RC of the model based on DD and V_FD_ was equal to 100 %. Also, the statistical indexes in V_BD_ were equal to RC = 99 % and predicted RC = 93 %.

Equation (16) demonstrates the prediction model of DD based on effective features with an acceptable RC amount. Likewise, According to Table 2, it can be concluded that all parameters have p-values <0.0001, and they are significant. Besides, because of error values (Sum of Squares and Mean Squares), it can be seen that w, Ad, and D are more important than other ones.

(16) $$\text{DD}=0.010049+0.021471\times W-3.21922\times 10^{-3}\times \mu -0.054646\times A_{d}-0.17489\times \text{Ca}+1.05915\times D-6.03077\times 10^{-7}\times W\times \mu -9.34552\times 10^{-6}\times W\times A_{d}+1.65384\times 10^{-4}\times W\times \text{Ca}-2.76326\times 10^{-5}\times W\times D-4.64232\times 10^{-6}\times \mu \times A_{d}+1.22709\times 10^{-4}\times \mu \times \text{Ca}-1.92364\times 10^{-5}\times \mu \times D-2.45005\times 10^{-4}\times A_{d}\times \text{Ca}+4.11985\times 10^{-5}\times A_{d}\times D-2.21949\times 10^{-3}\times \text{Ca}\times D$$

**Table 2:** The ANOVA assessment as per DD function.

**Source**	**Sum of Squares**	**Mean Square**	**F Value**	**p-value**
**Model**	0.073812	0.004921	63660000	<0.0001
**A-w**	3.16E-06	3.16E-06	63660000	<0.0001
B−μ	1.71E-06	1.71E-06	63660000	<0.0001
**C-Ad**	6.63E-05	6.63E-05	63660000	<0.0001
**D-Ca**	2.78E-06	2.78E-06	63660000	<0.0001
**E-D**	0.001868	0.001868	63660000	<0.0001
**AB**	0	0		
**AC**	3.4E-13	3.4E-13	63660000	<0.0001
**AD**	0	0		
**AE**	0	0		
**BC**	4.58E-13	4.58E-13	63660000	<0.0001
**BD**	0	0		
**BE**	0	0		
**CD**	5.88E-13	5.88E-13	63660000	<0.0001
**CE**	0	0		
**DE**	8.92E-13	8.92E-13	63660000	<0.0001
**Residual**	0	0		
**Cor Total**	0.073812			

**Fig. 6:**
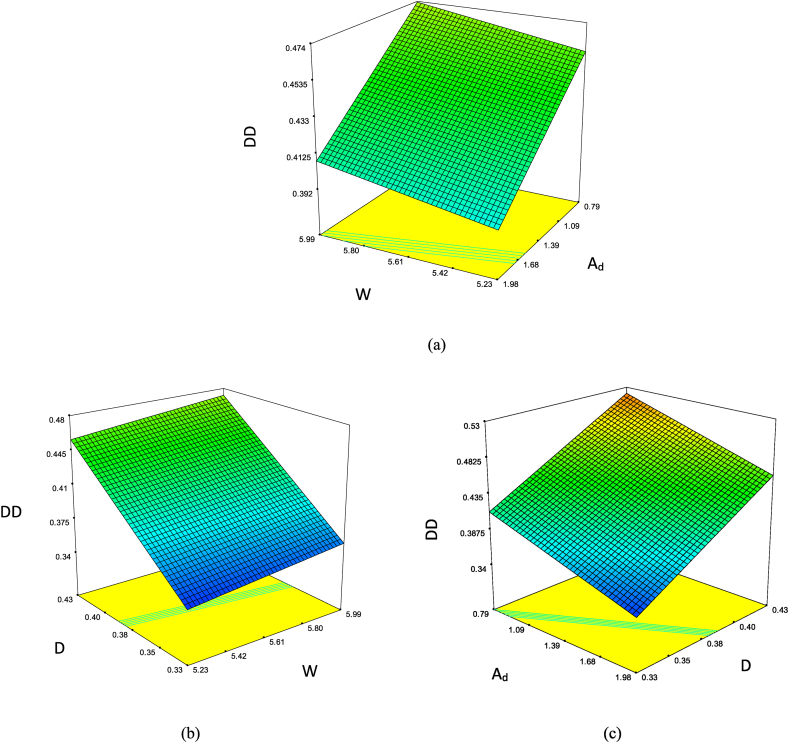
The sensitive analysis of the most effective features based on the DD function (a–c).

**Fig. 7:**
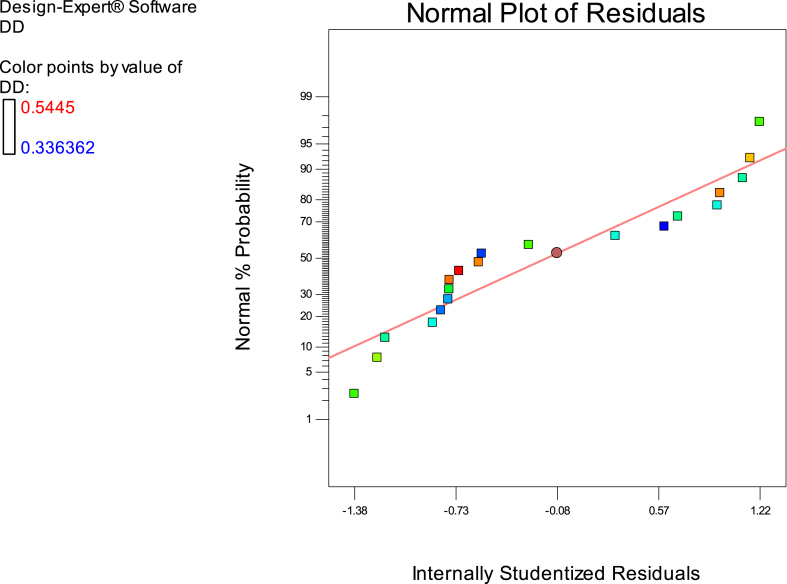
The normal diagram data analysis of effective parameters in this study as per DD response.

The sensitive analysis of the most effective parameters calibration is demonstrated in Fig. 6. Considering Fig. 6(a–c), it can be proven that (A_d_) is more significant than (W) and also (D > W). Meanwhile, the slope of D fluctuations is higher than that of Ad, and therefore, D is the most effective parameter. Finally, Fig. 7 illustrates that the applied experiments follow the normal distribution.

**Fig. 8:**
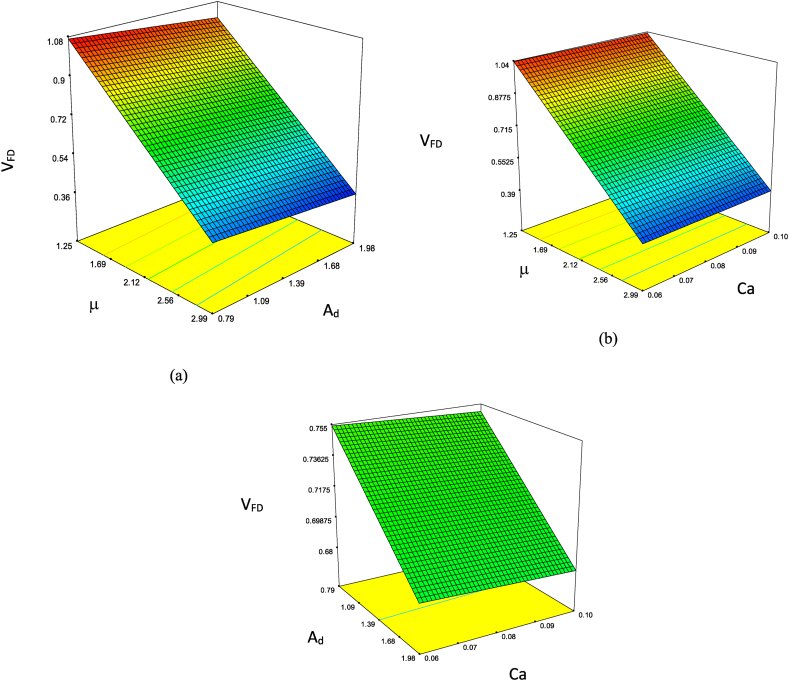
The sensitive analysis of the most significant factors in this investigation is based on V_FD_ (a–c).

**Fig. 9:**
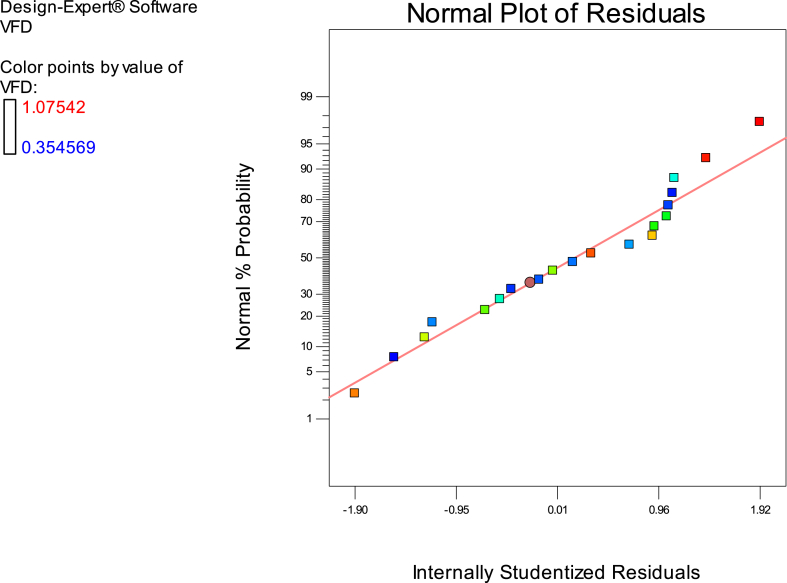
Illustrates the data analysis diagram of relevant parameters' normal distribution in VDF.

Equation (17) showcases the predictive model for V_FD_, reliant on effective features with a suitable RC quantity. Correspondingly, as indicated in Table 3, it can be deduced that all parameters possess p-values less than 0.0001, signifying their statistical significance. Furthermore, the error metrics, such as the Sum of Squares and Mean Squares, indicate that μ, A_d_, and Ca hold greater significance in comparison to other parameters. The sensitivity analysis of these paramount parameters is visualized in Fig. 8. Analyzing Fig. 8(a–c) reveals that the viscosity ratio outweighs A_d_, and the viscosity ratio holds a higher value than Ca. In contrast, the amplitude of fluctuations in A_d_ surpasses that of Ca, implying that the viscosity ratio is the most influential parameter. Lastly, Fig. 9 demonstrates the adherence of applied experiments to a normal distribution pattern.

(17) $$V_{\text{FD}}=1.49001+0.017323\times W-0.36688\times \mu -0.059225\times A_{d}-0.11120\times \text{Ca}-3.17766\times 10^{-3}\times D+3.75802\times 10^{-6}\times W\times \mu -3.50092\times 10^{-6}\times W\times A_{d}-2.12862\times 10^{-4}\times W\times \text{Ca}+7.32406\times 10^{-5}\times W\times D-1.13154\times 10^{-6}\times \mu \times A_{d}+2.92090\times 10^{-5}\times \mu \times \text{Ca}-1.52351\times 10^{-4}\times \mu \times D-1.18258\times 10^{-4}\times \text{Ad}\times \text{Ca}+3.83922\times 10^{-5}\times A_{d}\times D+5.63158\times 10^{-4}\times \text{Ca}\times D$$

**Table 3:** The ANOVA assessment as per V_FD_ function.

**Source**	**Sum of Squares**	**Mean of Squares**	**F Value**	**p-value**
**Model**	1.247112	0.083141	63660000	<0.0001
**A-W**	2.06E-06	2.06E-06	63660000	<0.0001
B−μ	0.022167	0.022167	63660000	<0.0001
**C-A**_**d**_	7.77E-05	7.77E-05	63660000	<0.0001
**D-Ca**	1.15E-06	1.15E-06	63660000	<0.0001
**E-D**	1.21E-08	1.21E-08	63660000	<0.0001
**AB**	0	0		
**AC**	0	0		
**AD**	0	0		
**AE**	0	0		
**BC**	0	0		
**BD**	0	0		
**BE**	0	0		
**CD**	0	0		
**CE**	0	0		
**DE**	0	0		
**Residual**	0	0		
**Cor Total**	1.247112

**Fig. 10:**
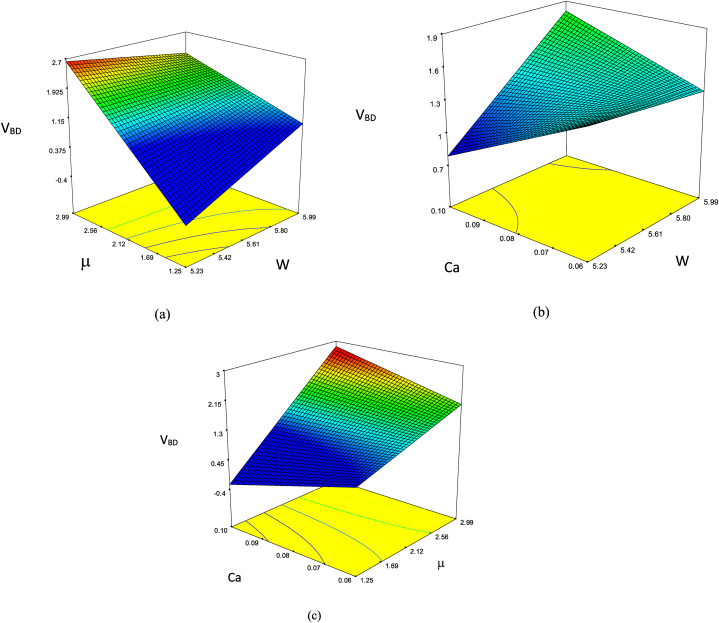
The dual sensitive evaluation of the most important features according to V_BD_ function.

**Fig. 11:**
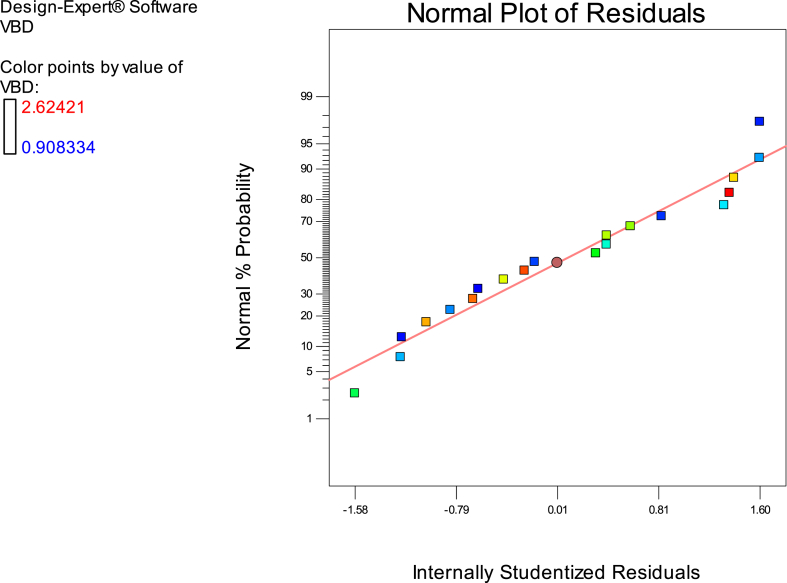
Normal plot of residuals based on V_BD_ function.

Equation (18) presents the predictive framework for V_BD_, utilizing significant features alongside an appropriate RC level. Similarly, as detailed in Table 4, it becomes apparent that the p-values of the viscosity ratio are below 0.0001, underscoring their statistical importance. The sensitivity analysis of these critical parameters is graphically depicted in Fig. 10. Examination of Fig. 10(a–c) underscores the dominance of the viscosity ratio over the other features in the experimental design. Fig. 11 depicts the alignment of executed experiments with a pattern of normal distribution.

(18) $$V_{\text{BD}}=-56.57042+9.05591\times W+9.56758\times \mu +0.78769\times A_{d}-285.04587\times \text{Ca}+162.58262\times D-1.48326\times W\times \mu +0.42921\times W\times A_{d}+41.98542\times W\times \text{Ca}-24.86951\times W\times D-0.20628\times \mu \times A_{d}+32.96073\times \mu \times \text{Ca}-6.53905\times \mu \times D-10.05758\times A_{d}\times \text{Ca}-4.95905\times A_{d}\times D-24.59576\times \text{Ca}\times D$$

**Table 4:** The ANOVA evaluations as per V_BD_ function.

**Source**	**Sum of Squares**	**Mean Square**	**F Value**	**P value**
**Model**	6.925541	0.461703	673.38	<0.0001
**A-W**	0.002312	0.002312	3.37	0.1402
B−μ	0.228739	0.228739	333.61	<0.0001
**C-A**_**d**_	0.000156	0.000156	0.227	0.6584
**D-Ca**	0.000706	0.000706	1.03	0.3677
**E-D**	0.000194	0.000194	0.28	0.6228
**AB**	0.012112	0.012112	17.66	0.0137
**AC**	0.000718	0.000718	1.04	0.3641
**AD**	0.005954	0.005954	8.68	0.0421
**AE**	0.006077	0.006077	8.86	0.0409
**BC**	0.000904	0.000904	1.32	0.3148
**BD**	0.012927	0.012927	18.85	0.0122
**BE**	0.017649	0.017649	25.74	0.0071
**CD**	0.000991	0.000991	1.44	0.2956
**CE**	0.000633	0.000633	0.92	0.3909
**DE**	0.00011	0.00011	0.16	0.7097
**Residual**	0.002743	0.000686		
**Cor Total**	6.928283

Table 5 illustrates the optimal conditions of effective factors in the multi-objective optimization process. In the optimization system, DD, V_FD_, and V_BD_ are minimized, minimized, and maximized, respectively. The suggested amounts for the optimization practice are declared in Table 5.

**Table 5:** The suggested optimal condition in a multi-objective optimization system.

**Number**	**W**	μ	**A**_**d**_	**Ca**	**D**	**DD**	**V**_**FD**_	**V**_**BD**_	**Desirability**
**1**	5.42	2.99	1.98	0.1	0.32	0.333212	0.357648	2.812345	0.998574
**2**	5.45	2.99	1.98	0.09	0.32	0.336359	0.358728	2.798172	0.998073
**3**	5.52	2.99	1.98	0.1	0.32	0.332373	0.359561	3.000754	0.997686
**4**	5.52	2.99	1.98	0.1	0.32	0.332438	0.359563	2.972593	0.997685

### 4.2 AI-based control system applications

**Fig. 12:**
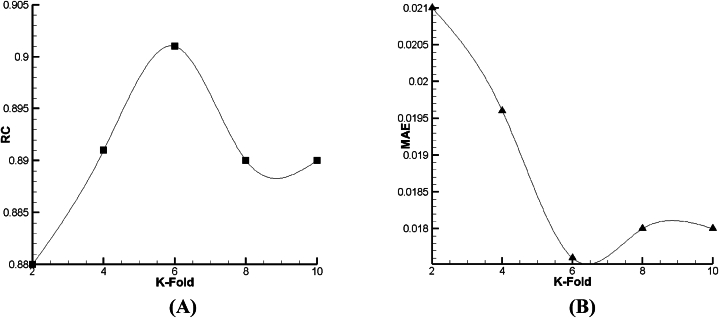
The tuning process of Lazy-IBK for prediction of DD based on (A) RC and (B) MAE.

**Fig. 13:**
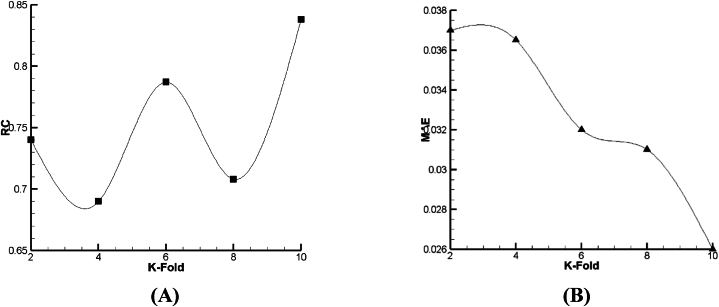
The calibration of Meta-Bagging for forecasting of DD based on (A) RC and (B) MAE.

**Fig. 14:**
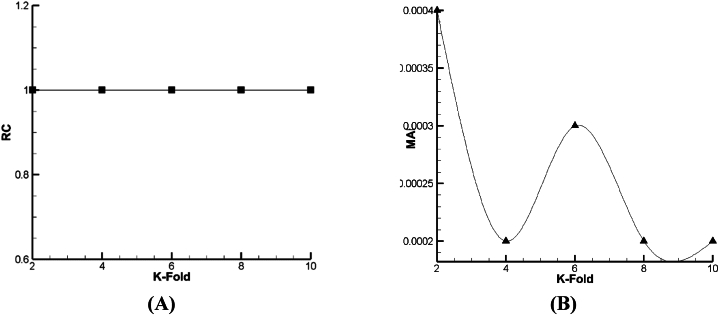
The calibration of function. SMOreg for estimation of DD based on (A) RC and (B) MAE.

**Fig. 15:**
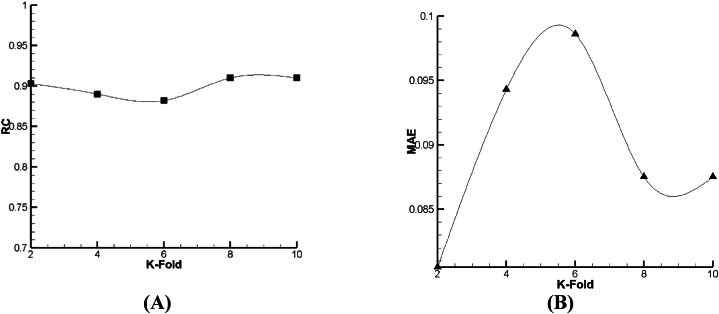
The tuning process of Lazy-IBK for prediction of V_FD_ based on (A) RC and (B) MAE.

For prediction of droplet distance (DD) as a function, the outcomes of lazy-IBK, Meta Bagging, and SMOreg computations are reported in Fig. 12, Fig. 13, Fig. 14. As per Fig. 13, Fig. 14, Fig. 15 a and b, it can be seen that the 6-fold is the best condition for the prediction of DD based on lazy-IBK algorithm. By the model, as per Tables 1 and it is clear that for the lazy algorithm, the best prediction of DD occurred with Regression Coefficient (RC) and MAE equal to 0.901 and 0.0176, respectively. Meanwhile, regarding the Meta-Bagging algorithm (Fig. 12, Fig. 13, Fig. 14), the best estimation is done 10-fold, with RC = 0.838 and MAE = 0.026. Finally, for the SMOreg algorithm, the most appropriate forecasting is executed in RC = 1 and MAE = 0.0002 as an output of 8-fold (Fig. 15).

**Fig. 16:**
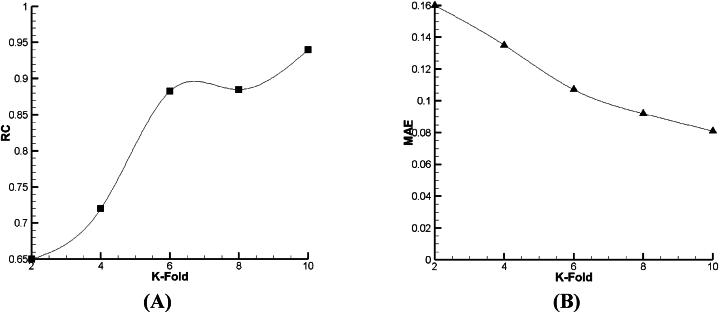
The calibration of Meta-Bagging for forecasting of V_FD_ based on (A) RC and (B) MAE.

**Fig. 17:**
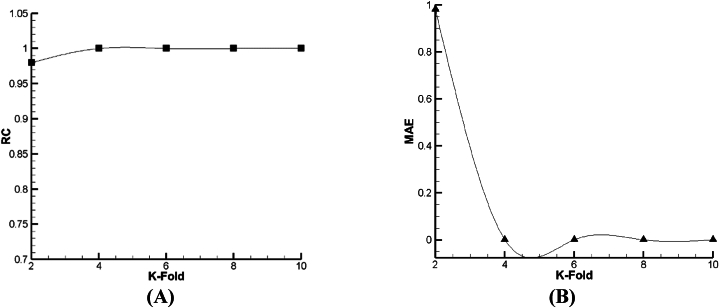
The calibration of function. SMOreg for estimation of V_FD_ based on (A) RC and (B) MAE.

According to Fig. 15, Fig. 16, Fig. 17, it is demonstrated that for Lazy-IBK, Meta-Bagging, and SMOreg, the best prediction can be found in 8-fold (RC = 0.91, MAE = 0.0875), 8-fold (RC = 0.885, MAE = 0.092), and 8-fold (RC = 1, MAE = 0.0006), respectively.

**Fig. 18:**
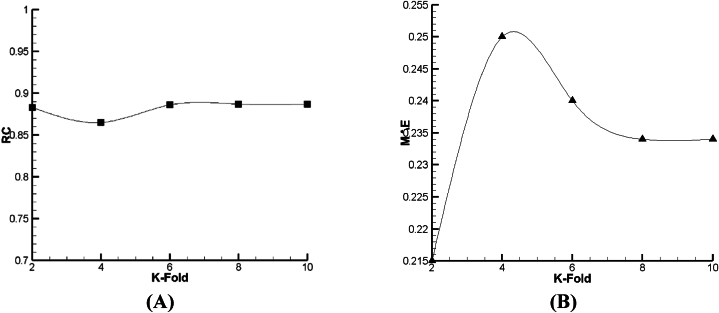
The tuning process of Lazy-IBK for prediction of V_BD_ based on (A) RC and (B) MAE.

**Fig. 19:**
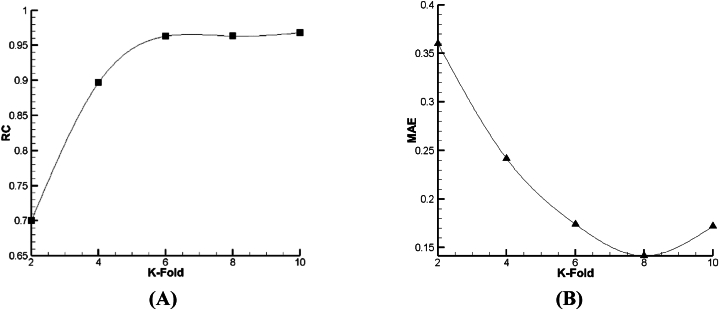
The calibration of Meta-Bagging for forecasting of V_BD_ based on (A) RC and (B) MAE.

**Fig. 20:**
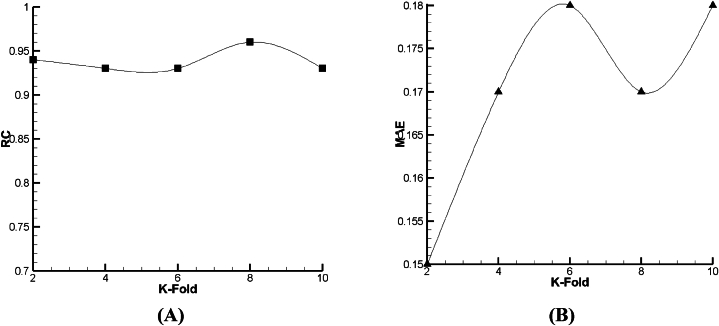
The calibration of function. SMOreg for estimation of V_BD_ based on (A) RC and (B) MAE.

As given in Fig. 18, Fig. 19, Fig. 20, it is clear that such as V_FD_, in V_BD_ prediction, the best condition in all the declared algorithms occurs in the 8th fold—the best RC of Lazy-IBK, Meta-Bagging, and SMOreg are equal to 0.88 (MAE = 0.234), 0.96 (MAE = 0.1414), and 0.96 (MAE = 0.17), correspondingly.

For holistic judgment about the performance of different algorithms, it should be mentioned that based on both MAE and RC (Table 6), for the prediction of DD, V_FD_, and V_BD_, the best algorithms include function. SMOreg, Lazy-IBK, and Meta-Bagging, respectively.

**Table 6:** The statistical outputs of artificial intelligence based on DD, V_FD_, and V_BD_ prediction.

	**RC**			**MAE**			**RMSE**		
	**DD**	V_FD_	V_BD_	**DD**	V_FD_	V_BD_	**DD**	V_FD_	V_BD_
**Lazy-IBK**	0.901	0.91	0.887	0.0176	0.087	0.234	0.028	0.105	0.283
**Meta-Bagging**	0.838	0.948	0.96	0.026	0.081	0.1414	0.0442	0.094	0.175
**Function. SMOreg**	1	1	0.93	0.0002	0.0006	0.1786	0.0003	0.0009	0.2101

This section of the research unveils a profound application of the Function framework, showcasing its versatility and significance. The utilization of the SMOreg algorithm stands out prominently, offering a powerful tool for predicting DD and V_FD_. The algorithm's efficacy in this regard underscores its value in tackling complex predictive tasks.

Furthermore, within the realm of predictive modeling, the choice of technique plays a pivotal role. In this context, the Meta-Bagging technique emerges as an exceptionally fitting approach for estimating V_BD_, i.e., a task that inherently demands robust and accurate methodologies. Through meticulous comparison with alternative techniques, the superiority of Meta-Bagging becomes evident as it consistently delivers precise estimations and demonstrates a remarkable ability to handle the intricate nuances of V_BD_.

In essence, this research segment not only underscores the practicality of the Function framework and the proficiency of the SMOreg algorithm but also sheds light on the strategic selection of Meta-Bagging, which is the prime technique for unraveling the complexities of V_BD_ estimation. The amalgamation of these techniques not only advances the field but also serves as a testament to the thoughtful and meticulous approach undertaken in this study.

## 5 Conclusion

In this paper, our investigation into the dynamics of droplet coalescence within a sudden expansion microfluidic system has yielded significant insights and advancements. By employing a combination of fundamental fluid dynamics equations, boundary integral methods, and cutting-edge machine learning techniques, we have tackled the complex interplay of droplet behaviors in microscale environments.

One key aspect of our study involved the utilization of the boundary integral method to transform the Brinkman partial differential equation into a self-consistent integral equation, which was then efficiently solved using the boundary element method. This approach provided a detailed understanding of droplet coalescence mechanics, particularly concerning deformable droplets as they interact within the microfluidic channel.

Furthermore, our integration of machine learning techniques, including Lazy-IBK, SMOreg, and Meta Bagging, allowed for the prediction of essential cost functions such as droplet-droplet distance (DD), the velocity of the front droplet (V_FD_), and velocity of the back droplet (V_BD_). The high predictive accuracy achieved through these methods indicates their potential for enhancing experimental efficiency and reducing errors, particularly in industrial applications.

There are several promising avenues for future research in this domain. Firstly, expanding our investigations to encompass multi-phase systems, such as emulsions or foams, could offer deeper insights into the behavior of multiple droplets interacting within microfluidic channels. Understanding the dynamics of such systems could have significant implications for various industries, including pharmaceuticals and food processing.

Secondly, exploring advanced machine learning techniques, such as deep learning and neural networks, holds promise for further enhancing predictive capabilities. These methods have the potential to uncover intricate patterns and relationships within experimental data, facilitating more accurate predictions and optimizations in microfluidic research and industrial applications alike.

In conclusion, our study represents a significant step forward in understanding and optimizing droplet coalescence dynamics in microchannels. By integrating numerical methods, machine learning algorithms, and experimental optimization techniques, we have contributed to advancing the field of microfluidics, paving the way for more efficient and precise control over fluidic processes in diverse applications.

The values of A_d_ and D are the most significant factors affecting DD, with the viscosity ratio having the least impact. For droplet velocity, viscosity, and channel width are the most influential parameters, while the initial distance and Ca have the least effect.

The relationship between the initial and final droplet distances (D and DD) is linear. Notably, when Ad exceeds 1, the droplet-droplet distance remains almost unchanged as the droplet area increases. The viscosity ratio has minimal impact on droplet distance, but an increase in the viscosity ratio results in a 16 % decrease in droplet velocity.

To present a comprehensive evaluation of the performance of various algorithms, it is important to note that, according to both MAE and RC, the top algorithms for predicting DD, V_FD_, and V_BD_ are function, SMOreg, Lazy-IBK, and Meta-Bagging, respectively.

## CRediT authorship contribution statement

**Seyed Morteza Javadpour:** Supervision, Software, Methodology. **Erfan Kadivar:** Writing – original draft, Resources, Investigation. **Zienab Heidary Zarneh:** Writing – original draft, Conceptualization. **Ebrahim Kadivar:** Resources, Conceptualization. **Mohammad Gheibi:** Visualization, Methodology.

## Availability of data and materials

The datasets used and analyzed during the current study are available from the corresponding author upon reasonable request.

## Declaration of competing interest

The authors declare that they have no known competing financial interests or personal relationships that could have appeared to influence the work reported in this paper.
